# Supplementary material for: Effect of growing regions on morphological characteristics, protein subfractions, rumen degradation and molecular structures of various whole-plant silage corn cultivars
Source: PLoS One. 2024 Jan 11;19(1):e0282547. doi: 10.1371/journal.pone.0282547 (PMC10783724; doi:10.1371/journal.pone.0282547)
Supplement: Attachment — Submitted filename: PONE-D-23-04492.pdf [file pone.0282547.s003.pdf]

PLOS ONE

Effect of growing regions on morphological characteristics, protein nutrition, rumen degradation and molecular structures of various whole-plant silage corn cultivars

--Manuscript Draft--

**Manuscript Number:**	PONE-D-23-04492
**Article Type:**	Research Article
**Full Title:**	Effect of growing regions on morphological characteristics, protein nutrition, rumen degradation and molecular structures of various whole-plant silage corn cultivars
**Short Title:**	Differences on protein nutrition, rumen degradation and molecular structures of various silage corn cultivars
**Corresponding Author:**	Xinyue Zhang<br>Northeast Agricultural University<br>Harbin, CHINA
**Keywords:**	whole-plant silage corn; morphological characteristic; protein chemical value; in situ degradation; molecular structure
**Abstract:**	Little information exists on the variation of morphological characteristics, nutritional value, ruminal degradability, and molecular structural makeup of diverse whole-plant silage corn (WPSC) cultivars among different growing regions. This study investigated the between-regions discrepancies in five widely used WPSC cultivars in China (FKBN, YQ889, YQ23, DK301 and ZD958) in terms of 1) morphological characteristics; 2) crude protein (CP) chemical profile; 3) Cornell Net Carbohydrate and Protein System (CNCPS) CP subfractions; 4) in situ CP degradation kinetics; and 5) CP molecular structures. Our results revealed that significant difference were observed on growing region and WPSC cultivar interaction for all estimated morphological characteristics ($P < 0.001$), CP chemical profile ($P < 0.001$), CNCPS subfractions ($P < 0.001$) and CP molecular structural features ($P < 0.05$). Except ear weight ($P = 0.18$), all measured morphological characteristics varied among different growing regions ($P < 0.001$). Besides, WPSC cultivars planted in different areas had remarkably different ($P < 0.01$) CP chemical profiles and CNCPS subfractions. All spectral parameters of protein primary structure of WPSC differed ($P < 0.05$) due to the various growing regions, except amide II area ($P = 0.28$). Finally, the area ratio of amide I to II was negatively correlated with the contents of soluble CP ($\delta = -0.66$; $P = 0.002$), CP ($\delta = -0.61$; $P = 0.006$), non-protein N ($\delta = -0.56$; $P = 0.004$) and acid detergent insoluble CP ($\delta = -0.43$; $P = 0.008$), in conjunction with positively correlated with moderately degradable CP (PB1; $\delta = 0.58$; $P = 0.01$). In conclusion, the current study suggested that even for the same WPSC cultivar, the morphological characteristics, protein nutritional values and rumen degradability significantly varied among different grown regions due to distinguished molecular structures.
**Order of Authors:**	Xinyue Zhang
	Nazir Ahmad Khan
	Enyue Yao
	Fanlin Kong
	Ming Chen
	Rifat Ullah Khan
	Xin Liu
	Yonggen Zhang
	Hangshu Xin
**Additional Information:**	
**Question**	**Response**
**Financial Disclosure**	This research was funded by the National Natural Science Foundation of China (Grant

Powered by Editorial Manager® and ProduXion Manager® from Aries Systems Corporation

Enter a financial disclosure statement that describes the sources of funding for the work included in this submission. Review the submission guidelines for detailed requirements. View published research articles from *PLOS ONE* for specific examples.<br><br>This statement is required for submission and **will appear in the published article** if the submission is accepted. Please make sure it is accurate.<br><br>**Unfunded studies**<br>Enter: *The author(s) received no specific funding for this work.*<br><br>**Funded studies**<br>Enter a statement with the following details:<br>• Initials of the authors who received each award<br>• Grant numbers awarded to each author<br>• The full name of each funder<br>• URL of each funder website<br>• Did the sponsors or funders play any role in the study design, data collection and analysis, decision to publish, or preparation of the manuscript?<br>• **NO** - Include this sentence at the end of your statement: *The funders had no role in study design, data collection and analysis, decision to publish, or preparation of the manuscript.*<br>• **YES** - Specify the role(s) played.<br><br>* typeset	No. 31702135) and China Agriculture Research System of MOF and MARA.
**Competing Interests**<br><br>Use the instructions below to enter a competing interest statement for this submission. On behalf of all authors, disclose any competing interests that could be perceived to bias this work—acknowledging all financial support and any other relevant financial or non-financial competing interests.<br><br>This statement is required for submission and **will appear in the published article** if	The authors have declared that no competing interests exist.

the submission is accepted. Please make sure it is accurate and that any funding sources listed in your Funding Information later in the submission form are also declared in your Financial Disclosure statement.<br><br>View published research articles from *PLOS ONE* for specific examples.<br><br>**NO authors have competing interests**<br><br>Enter: *The authors have declared that no competing interests exist.*<br><br>**Authors with competing interests**<br><br>Enter competing interest details beginning with this statement:<br><br>*I have read the journal's policy and the authors of this manuscript have the following competing interests: [insert competing interests here]*<br><br>* typeset	
**Ethics Statement**<br><br>Enter an ethics statement for this submission. This statement is required if the study involved:<br><br>• Human participants<br>• Human specimens or tissue<br>• Vertebrate animals or cephalopods<br>• Vertebrate embryos or tissues<br>• Field research<br><br>Write "N/A" if the submission does not require an ethics statement.<br><br>General guidance is provided below. Consult the submission guidelines for detailed instructions. **Make sure that all information entered here is included in the Methods section of the manuscript.**	The study was approved by the Ethical Committee of the College of Animal Science and Technology, Northeast Agriculture University for animal experiment (NEAU-[2011]-9).

Powered by Editorial Manager® and ProduXion Manager® from Aries Systems Corporation

**Format for specific study types**<br><br>**Human Subject Research (involving human participants and/or tissue)**<br>• Give the name of the institutional review board or ethics committee that approved the study<br>• Include the approval number and/or a statement indicating approval of this research<br>• Indicate the form of consent obtained (written/oral) or the reason that consent was not obtained (e.g. the data were analyzed anonymously)<br><br>**Animal Research (involving vertebrate animals, embryos or tissues)**<br>• Provide the name of the Institutional Animal Care and Use Committee (IACUC) or other relevant ethics board that reviewed the study protocol, and indicate whether they approved this research or granted a formal waiver of ethical approval<br>• Include an approval number if one was obtained<br>• If the study involved *non-human primates*, add *additional details* about animal welfare and steps taken to ameliorate suffering<br>• If anesthesia, euthanasia, or any kind of animal sacrifice is part of the study, include briefly which substances and/or methods were applied<br><br>**Field Research**<br><br>Include the following details if this study involves the collection of plant, animal, or other materials from a natural setting:<br>• Field permit number<br>• Name of the institution or relevant body that granted permission	
**Data Availability**<br><br>Authors are required to make all data underlying the findings described fully available, without restriction, and from the time of publication. PLOS allows rare exceptions to address legal and ethical concerns. See the PLOS Data Policy and FAQ for detailed information.	Yes - all data are fully available without restriction

A Data Availability Statement describing where the data can be found is required at submission. Your answers to this question constitute the Data Availability Statement and **will be published in the article**, if accepted.<br><br>**Important:** Stating 'data available on request from the author' is not sufficient. If your data are only available upon request, select 'No' for the first question and explain your exceptional situation in the text box.<br><br>Do the authors confirm that all data underlying the findings described in their manuscript are fully available without restriction?	
**Describe where the data may be found in full sentences. If you are copying our sample text, replace any instances of XXX with the appropriate details.**<br><br>• If the data are **held or will be held in a public repository**, include URLs, accession numbers or DOIs. If this information will only be available after acceptance, indicate this by ticking the box below. For example: *All XXX files are available from the XXX database (accession number(s) XXX, XXX.).*<br>• If the data are all contained **within the manuscript and/or Supporting Information files**, enter the following: *All relevant data are within the manuscript and its Supporting Information files.*<br>• If neither of these applies but you are able to provide **details of access elsewhere**, with or without limitations, please do so. For example:<br><br>*Data cannot be shared publicly because of [XXX]. Data are available from the XXX Institutional Data Access / Ethics Committee (contact via XXX) for researchers who meet the criteria for access to confidential data.*<br><br>*The data underlying the results presented in the study are available from (include the name of the third party*	The data underlying the results presented in the study are available from the corresponding author upon reasonable request

and contact information or URL).<br>• This text is appropriate if the data are owned by a third party and authors do not have permission to share the data.<br><br>* typeset	
Additional data availability information:	

Effect of growing regions on morphological characteristics, protein nutrition, rumen degradation and molecular structures of various whole-plant silage corn cultivars

Xinyue Zhang[1,2Y], Nazir Ahmad Khan[3Y], Enyue Yao[1,4], Fanlin Kong[2], Ming Chen[5], Rifat Ullah Khan, Xin Liu[1,6], Yonggen Zhang[1], Hangshu Xin[1*], with the Lorem Ipsum Consortium¶

1 College of Animal Science and Technology, Northeast Agricultural University, Harbin, 150030, China

2 State Key Laboratory of Animal Nutrition, Beijing Engineering Technology Research Center of Raw Milk Quality and Safety Control, College of Animal Science and Technology, China Agricultural University, Beijing, 100193, China

3 Department of Animal Nutrition, The University of Agriculture Peshawar, Pakistan

4 Gushi Biological Group, Harbin, 150030, China

5 College of Agronomy and Biotechnology, National Maize Improvement Center of China, China Agricultural University, Beijing, 100091, China

6 Wellhope Shenyang Ruminant Feed Company Limited, Shenyang, 110164, China

Y These authors contributed equally to this work.

* correspondingauthor: hangshuxin@neau.edu.cn

Abstract

Little information exists on the variation of morphological characteristics, nutritional value, ruminal degradability, and molecular structural makeup of diverse whole-plant silage corn (WPSC) cultivars among different growing regions. This study investigated the between-regions discrepancies in five widely used WPSC cultivars in China (FKBN, YQ889, YQ23, DK301 and ZD958) in terms of 1) morphological characteristics; 2) crude protein (CP) chemical profile; 3) Cornell Net Carbohydrate and Protein System (CNCPS) CP subfractions; 4) *in situ* CP degradation kinetics; and 5) CP molecular structures. Our results revealed that significant difference were observed on growing region and WPSC cultivar interaction for all estimated morphological characteristics ($P < 0.001$), CP chemical profile ($P < 0.001$), CNCPS subfractions ($P < 0.001$) and CP molecular structural features ($P < 0.05$). Except ear weight ($P = 0.18$), all measured morphological characteristics varied among different growing regions ($P < 0.001$). Besides, WPSC cultivars planted in different areas had remarkably different ($P < 0.01$) CP chemical profiles and CNCPS subfractions. All spectral parameters of protein primary structure of WPSC differed ($P < 0.05$) due to the various growing regions, except amide II area ($P = 0.28$). Finally, the area ratio of amide I to II was negatively correlated with the contents of soluble CP ($\delta = -0.66$; $P = 0.002$), CP ($\delta = -0.61$; $P = 0.006$), non-protein N ($\delta = -0.56$; $P = 0.004$) and acid detergent insoluble CP ($\delta = -0.43$; $P = 0.008$), in conjunction with positively correlated with moderately degradable CP (PB_1; $\delta = 0.58$; $P = 0.01$). In conclusion, the current study suggested that even for the same WPSC cultivar, the morphological characteristics, protein nutritional values and rumen degradability significantly varied among different grown regions due to distinguished molecular structures.

Author summary

As the major roughage source, whole plant silage corn plays an essential role in ruminant feed industry. The quality and quantity of it largely influenced by environmental and climate conditions except genetic factors. However, there was limited information to systematically analyze whole plant silage corn from morphological characteristics, nutritional components, rumen degradation to its inherent molecular structures. Thus, this study was conducted to investigate the discrepancies of various silage-corn cultivars grown in different regions from internal structure to phenotype based novel technology - fourier transform infrared spectroscopy.

Introduction

Globally, whole plant silage corn (WPSC) is a major ingredient in dairy total mixed ration (TMR) under most dietary regimes, owing to its high and stable biomass yield under a wide range of environment and soil conditions, in conjunction with its high contents of total digestible nutrients and metabolizable energy [1-3]. Moreover, the inclusion of corn silage in TMR increased dry matter (DM) and metabolizable energy intakes as well as milk yield of dairy cows [4]. Because of these great qualities, WPSC contributed up to 42% of TMR for high-producing dairy cows [5]. Thus, optimizing the biomass yield and nutritional qualities of WPSC is a long-term goal for dairy industry to meet the nutritional requirements of high production dairy cows on sustainable basis.

The exception of genotype and harvest maturity, the yield and nutritional quality of WPSC are highly influenced by environmental conditions [6-8]. For instance, high growing temperature reduced the digestibility of corn silage, which is a result of increased lignin content in stovers and decreased starch content in cobs [9-10]. A close association was observed between soil moisture and plants' nutrients distribution in eight cultivars of napiergrass [10]. Moreover, Temime et al. [9] also reported that besides genetic factors, the proportion of volatile constituents in Chétouiolive oils was strongly influenced by growing regions. Similarly, the glycosidase inhibitory rate in mulberry leaves ranged from 142 to 845 g/kg between different cultivate regions were observed [11].

Although lower content of crude protein on average (CP; 74 g/kg DM) was observed in corn silage, it significantly contributed to the overall supply of metabolizable protein for dairy cows because of high consumption [3]. Moreover, rumen protein degradability and overall supply of metabolizable protein to dairy cows were strongly influenced by variations of CP chemical profiles and protein inherent molecular structures in feedstuffs [4, 12]. Recent studies suggested that the molecular structure of roughages could be revealed by advanced vibrational molecular spectroscopy techniques, for example, attenuated total reflectance - Fourier transform infrared spectroscopy (ATR-FTIR) [13-15]. Thus, it is possible to associate the protein chemical profiles and in situ degradation parameters with the specific inherent structures [16-19].

To our knowledge, no systematic study was conducted on morphological characteristics, CP rumen degradabilities and molecular structures of various WPSC cultivars planted in different growing regions. Therefore, in the present study, five widely utilized WPSC cultivars planted in five growing regions (Beijing, Urumchi, Cangzhou, Tianjin and Liaoyuan) in China were evaluated the differences on 1) morphological traits; 2) CP chemical profile; 3) Cornell Net Carbohydrate and Protein System (CNCPS) CP subfractions; 4) in situ CP degradation kinetics; and 5) protein molecular structures. The relationships between protein molecular structural parameters and CP chemical profile, CNCPS subfractions and ruminal degradation characteristics were also investigated. We hypothesized that the morphological

characteristics, CP chemical profile, and ruminal degradability may differ in WPSC cultivated in different growing regions, and those discrepancies in protein nutritional value and rumen degradability were correlated with their inherent protein molecular structures.

Materials and methods

Corn cultivars, growing regions, and crop production

Five WPCS cultivars widely utilized for production in China were selected for this study, including Fengkenbainuo (FKBN), Yaqing 889 (YQ889), Yuqing 23 (YQ23), Dongke 301 (DK301) and Zhengdan 958 (ZD958). All samples were grown in five different regions, which were Beijing (39°9' N, 116°3' E), Urumchi (43°8' N, 87°7' E), Cangzhou (38°3' N, 116°8' E), Tianjin (39°1' N, 117°2' E) and Liaoyuan (42°7' N, 125°5' E). In each region, these five cultivars were sown in three plots (5 m × 5 m) with seeding rate of 1400 kernels per plot, in conjunction with plant-to-plant distance of 35 cm and row to row distance of 60 cm. All plots in five growing regions were treated under the same practical management and agronomic practices during cultivation. Data on geographical and climate condition of five experimental regions were summarized in Table 1.

Table 1. Longitude and latitude, average annual temperature, and average annual precipitation of different growing regions of whole-plant silage corn cultivars.

Region	Longitude	Latitude	Temperature (˚C)	Precipitation (mm)
Beijing	E 116°3'	N 39°9'	July: 23～31	July: 97.5
			August: 21～30	August: 233.9
			September: 17～27	September: 2.8
			October: 7～15	October: 73.3
Urumchi	E 87°7'	N 43°8'	July: 21～31	July: 3.2
			August: 17～26	August: 14.5
			September: 11～22	September: 3.7
			October: 2～11	October: 8.8
Cangzhou	E 116°8'	N 38°3'	July: 24～32	July: 161.0
			August: 22～30	August: 124.7
			September: 18～29	September: 4.7
			October: 8～16	October: 138.6
Liaoyuan	E 125°5'	N 42°7'	July: 19～30	July: 231.8
			August: 17～27	August: 259.0
			September: 9～23	September: 16.7
			October: 0～12	October: 1.6
Tianjin	E 117°2'	N 39°1'	July: 25～32	July: 159.6
			August: 23～31	August: 160.4
			September: 19～29	September: 1.2
			October: 9～17	October: 101.9

Crop sampling and morphological measurements

Twenty plants (10 cm above the ground) from each plot were randomly harvested at kernel maturity stage of half milk line when targeted DM content was around 330 g/kg. The exterior 1 m area of each plot was excluded from sampling to ensure uniformity in the sampled plants. The physical parameters of WPCS including whole-plant height, whole-plant weight, stem diameter, ear height, ear weight, ear length, ear diameter, ear rows and kernel numbers in each row were measured and recorded at harvest. Then, the whole-plant samples were chopped to small particle size (1-2 cm), dried (at 60 °C for 24 h) and grounded through a 1.0 mm screen (FZ102, Taisite Instrument Co., Ltd, Tianjin, China) for analysis of chemical components and CNCPS compositions, or through a 2 mm screen for *in situ* incubation measurements, or through a 0.25 mm screen for molecular spectral analysis of protein primary and secondary structures.

Protein chemical analysis and subfractions composition

The WPSC samples were analyzed for CP content (official method 984.13, using a Kjeltec 2400 autoanalyzer; Foss Analytical A/S, Hillerφd, Denmark) according to AOAC [20]. The contents of soluble CP (SCP), neutral detergent insoluble CP (NDICP) and acid detergent insoluble CP (ADICP) were determined according to Licitra et al [21].

For estimating the difference of CP degradation in the rumen, the CP subfractions composition were determined according to the updated version (v 6.5) of Cornell Net Carbohydrate Protein System [CNCPS, 22-23]. A total of five subfractions quantified in this study were: PA_1 (non-protein N; ruminal degradation rate (k_d) of 2/h), PA_2 (soluble non-ammonia protein; k_d of 0.10-0.40/h), PB_1 (moderately degradable CP; k_d of 0.03-0.20/h), PB_2 (slowly degradable CP; k_d of 0.01-0.18/h) and PC (unavailable CP).

These CP subfractions were estimated from the chemical profiles according to the following mathematical models:

PA_1 (g/kg CP) = NPN (g/kg SCP) × 0.01 × SCP (g/kg CP)

PA_2 (g/kg CP) = SCP (g/kg SCP) - PA_1 (g/kg CP)

PB_1 (g/kg CP) = 1000 - PA_1 (g/kg CP) - PA_2 (g/kg CP) - PB_2 (g/kg CP) - PC (g/kg CP)

PB_2 (g/kg CP) = NDICP (g/kg CP) - ADICP (g/kg CP)

PC (g/kg CP) = ADICP (g/kg CP)

In situ rumen degradation

Three healthy and non-lactating Holstein Frisian dairy cows (550-600 kg body weight) fixed with permanent rumen fistula from the experimental base of Northeast Agricultural University were used for *in situ* incubation research. These animals were fed TMR with forage to concentrate ratio of 60:40, twice daily at 07:00 and 21:00 h. The TMR was composed of 428 g/kg Chinese wildrye, 158 g/kg corn silage, 132 g/kg corn, 74 g/kg corn gluten feed, 54 g/kg dried distiller grains, 49 g/kg corn germ meal, 37 g/kg wheat bran, 32 g/kg soybean meal, 21 g/kg cottonseed meal, 10 g/kg molasses beet, in conjunction with 5 g/kg minerals and vitamins.

Subsamples (ca. 7 g) were randomly incubated in sealed nylon bags (10 × 20 cm, pore size 40 μm) in rumen of three fistulated cows for 0, 4, 8, 12, 24, 36, 48 and 72 h, using the "gradual in/all out" schedule. Three replicate bags per sample from individual cow were used for every incubation time point. After incubation, all nylon bags were taken out of the rumen, washed with cold running tap water for six times, and then dried to constant weight in forced air over at 65 ℃. The dried residues from replicate bags of each sample were pooled according to the incubation time, then grounded and stored in plastic sealed bags for further analysis. Total three replicates were gained from three dairy cows. The parameters related to rumen degradation characteristics of CP were calculated according to Ørskov and McDonald [24].

Spectral data collection and analysis

Data on protein primary and secondary structures were collected at the Chemical Molecular Structure Analysis Laboratory located in Northeast Agriculture University (Harbin, China). Fourier transform infrared spectroscopy (FT/IR; Bruker ALPHA-T, Germany) was utilized for the molecular spectral analysis. The spectral data were obtained at the mid-infrared fingerprint region from ca. 4000 to 400 cm^{-1}, with 128 coadded scans at a spectral resolution of 4 cm^{-1}. Each sample was scanned five times. Data were collected at peak height and area of the spectral bands related to protein primary structural functional groups. Only the spectral intensities of amide I band (baseline: ca. 1712 - 1565 cm^{-1}; peak height: ca. 1639 cm^{-1}) and amide II band (baseline: ca. 1565 - 1489 cm^{-1}; peak height: ca. 1545 cm^{-1}) were analyzed. The fourier self-deconvolution (FSD) method and the second derivative function in OMNIC 8.2 software (Spectra Tech., Madison, WI, USA) were used to characterize the protein secondary structure in amide I region including protein α-helix (peak height: ca. 1656 cm^{-1}) and protein β-sheet (peak height: ca. 1634 cm^{-1}). The protein molecular structure spectral data quantified in this study were the peak height and area of protein amide I, amide II and their ratio, in conjunction with peak height of α-helix, β-sheet and their ratio.

To visualize the structural differences in protein structures, multivariate analysis, agglomerative hierarchical cluster analysis (CLA) and principal component analysis (PCA) were applied on the overall spectroscopic data within the protein amide I and amide II region (ca. 1712 - 1489 cm^{-1}), using Statistica 8.0 software (StatSoft Inc., Tulsa, OK, USA) [25].

Statistical analysis

The PROC MIXED procedure of SAS 9.4 was used to analyze data on the plant morphological characteristics, CP chemical profiles, CNCPS subfractions, *in situ* CP degradation kinetics and protein molecular structural parameters, the model used was: $Y_{ijkl} = \mu + C_i + R_j + C_i \times R_j + D_k + e_{ijkl}$, where Y_{ijkl} was an observation on the dependent variable ijkl, μ was the population mean of the variable, C_i was a fixed effect of corn cultivar (i = 5; FKBN, YQ889, YQ23, DK301 and ZD958), R_j was a fixed effect of growing region (j = 5; Beijing, Urumchi, Cangzhou, Tianjin and Liaoyuan), $C_i \times R_j$ was a fixed effect of interaction between factor C at level i and factor R at level j, D_k was the random effect of dairy cow, and eijkl was the model error.

The correlation between protein spectral parameters and protein chemical profile, CNCPS CP subfractions was well as *in situ* CP degradation data were analyzed by the Hmisc and Corrplot packages of R (version 4.0.2; R Foundation for Statistical Computing, Vienna, Austria). For all statistical analysis, significance was declared at $P < 0.05$, and tendency was declared at $0.05 < P < 0.10$.

Results and discussion

Effects of growing regions and cultivars on morphologicalcharacteristics of whole plant silage corn

The significant interactions between cultivar and growing region on morphologgical characteristics were showed in Table 2 ($P < 0.001$). Except ear weight ($P = 0.18$), all measured morphological characteristics varied ($P < 0.001$) among the growing regions. Plant height and weight, in conjunction with ear height and length were highest ($P < 0.05$) in WPSC cultivars grown in Urumchi, while whole plant height and weight, as well as ear length were lowest ($P < 0.05$) in Tianjin. Cultivars grown in Beijing represented greatest ($P < 0.05$) stem diameter, ear diameter and rows of kernel, whereas Urumchi showed the opposite condition ($P < 0.05$). Mean number of kernels in a row was highest ($P < 0.05$) in Tianjin and lowest ($P < 0.05$) in Beijing. Previous studies have reported that precipitation was one of the most influential abiotic factors for plant productivity [26], and drought stress generally contributed to delay in plant growth and development by decreasing cell elongation and reducing photosynthesis [27]. Moreover, soil moisture and growing

temperature were highly related to DM yields because they affected canopy and anatomical development of maize crop [10, 28]. Wang et al. [29] reported that the WPSC cultivar of Yunuo_7 had better leaf appearance rate at high growing temperature (37.5 ℃, southwest of China) than other maize cultivars adopted to northeast and north plain of China. Furthermore, there was an effect of solar radiation on growth components which were proportional to gross photosynthesis [30]. Results of Tollenaar et al. [31] showed that more than a quarter of DM yield of maize crop was attributable to solar brightening during 1984 to 2013 in the US Corn Belt. In addition, limited solar radiation during flowering reduced kernel number and kernel development of maize [32]. Above factors may partially explain the reasons for differences in morphological characteristics of WPSC cultivars from different growing regions observed in our study.

Table 2. Morphological measurements of silage-corn cultivars grown in different regions.

Cultivars	Regions	Whole plant		Stem	Ear					% Row kernels
		Height (cm)	Weight (kg)	Diameter (cm)	Height (cm)	Weight (kg)	Length (cm)	Diameter (cm)	Rows	
FKBN	*Beijing*	340.0	1.35	2.31	156.2	0.53	31.2	8.26	17.2	28.6
	Urumchi	320.1	2.26	1.66	168.7	0.84	29.7	5.72	13.8	40.8
	Cangzhou	317.8	0.89	1.80	151.3	0.33	27.5	5.59	13.2	28.8
	Tianjin	297.7	0.86	1.56	151.3	0.39	24.3	5.37	14.8	39.2
	Liaoyuan	293.2	1.01	2.00	149.8	0.43	26.6	6.28	16.0	42.2
YQ889	*Beijing*	316.0	0.96	2.09	152.5	0.36	24.4	5.92	16.6	30.1
	Urumchi	363.6	1.17	1.84	190.8	0.35	28.0	5.89	15.2	30.6
	Cangzhou	328.0	1.31	1.93	155.1	0.50	27.1	6.16	15.4	30.7
	Tianjin	254.2	1.99	1.75	122.0	0.72	23.5	5.96	15.6	33.6
	Liaoyuan	269.5	0.77	1.90	127.3	0.33	24.9	5.84	15.6	31.8
YQ23	*Beijing*	280.4	1.78	2.20	124.7	0.83	24.8	6.33	16.4	34.9
	Urumchi	345.5	0.96	1.59	186.3	0.31	29.3	5.64	13.8	24.2
	Cangzhou	330.4	1.15	1.68	154.8	0.44	25.1	5.86	16.2	32.8
	Tianjin	262.9	0.66	1.79	143.2	0.26	23.3	5.68	14.8	32.1
	Liaoyuan	296.0	1.21	2.12	154.0	0.44	26.1	6.04	14.4	37.8
DK301	*Beijing*	301.6	0.87	2.01	142.4	0.33	23.2	6.28	17.2	27.4
	Urumchi	351.0	1.32	1.97	182.8	0.42	31.6	6.34	14.0	33.7
	Cangzhou	282.9	1.93	1.71	131.8	0.80	23.4	6.24	14.6	32.2
	Tianjin	257.6	0.88	1.98	124.0	0.41	24.7	6.42	16.0	39.8
	Liaoyuan	298.6	1.02	1.69	158.1	0.33	27.3	5.72	14.8	34.8

ZD958	*Beijing*	271.8	0.80	1.87	137.6	0.33	24.0	6.15	15.8	26.7
	Urumchi	383.3	1.19	1.62	208.9	0.34	28.1	4.85	13.8	36.0
	Cangzhou	301.4	1.28	2.00	137.4	0.46	26.2	6.47	14.0	36.5
	Tianjin	340.5	1.08	2.32	185.3	0.53	23.9	6.41	17.7	40.3
	Liaoyuan	260.7	1.76	1.84	117.8	0.72	24.3	6.00	14.8	37.1
	SEM	69.04	0.82	0.42	46.54	0.34	4.85	1.16	2.36	9.36
Regions										
	Beijing	301.96^{c}	1.15^{b}	2.10^{a}	142.68^{b}	0.48^{ab}	25.52^{b}	6.59^{a}	16.64^{a}	29.54^{c}
	Urumchi	352.70^{a}	1.38^{a}	1.74^{c}	187.50^{a}	0.45^{b}	29.34^{a}	5.69^{c}	14.12^{d}	33.06^{b}
	Cangzhou	312.10^{b}	1.31^{a}	1.82^{bc}	146.08^{b}	0.51^{a}	25.86^{b}	6.06^{b}	14.68^{cd}	32.20^{b}
	Tianjin	282.58^{d}	1.09^{b}	1.88^{b}	145.16^{b}	0.46^{ab}	23.94^{c}	5.97^{b}	15.78^{b}	37.00^{a}
	Liaoyuan	283.60^{d}	1.15^{b}	1.91^{bc}	141.40^{b}	0.45^{b}	25.84^{b}	5.98^{b}	15.12^{bc}	36.74^{a}
	SEM	24.71	0.44	0.38	18.75	0.19	3.60	0.71	2.45	8.81
P-value	*Regions*	<0.001	<0.001	<0.001	<0.001	0.18	<0.001	<0.001	<0.001	<0.001
	Cultivar	<0.001	0.38	0.75	0.002	0.29	<0.001	0.001	0.35	0.001
	Region × Cultivar	<0.001	<0.001	<0.001	<0.001	<0.001	<0.001	<0.001	<0.001	<0.001

Note: FKBN = Fengkenbainuo; YQ 889 = Yaqing 889; YQ 23 = Yuqing 23; DK 301 = Dongke 301; ZD958 = Zhengdan 958.

Effects of growing regions and cultivars on protein chemical profiles and CNCPS of whole plant silage corn

The CNCPS was widely used for the evaluation of ruminant feedstuff [33-36]. In the present study, significant effect of cultivar and growing region interaction ($P < 0.001$) was observed for all measured CP chemical profiles and CNCPS subfractions (Table 3). WPSC cultivars grown in different areas exhibited remarkably different CP chemical profiles. For instance, the concentrations of ADICP (3.6 g/kg) and PC (49.3 g/kg) were lowest ($P < 0.05$) in WPSC cultivated in Urumchi. The results suggested that WPSC grown in Urumchi contained high proportion of protein available to animals, and implied that climate conditions (e.g. precipitation and growing temperature) could influence internal nutrient accumulations of WPSC. In agreement with our findings, previous study reported that the CP contents in maize and wheat were lower in rainy years [37]. High growth temperature markedly increased the lignin content of maize plants [11, 38], which can partly explain the lowest contents of ADICP (3.6 g/kg) and PC (49.3 g/kg) cultivated in Urumchi, where the lowest temperature was recorded among all five growing regions. As for the soluble protein contents, such as NPN and SCP, the WPSC planted in Beijing had higher ($P < 0.05$) content than other regions. Similarly, the CP content was greatest in Beijing (86.6 g/kg), followed

by Tianjin (85.7 g/kg), Cangzhou (78.2 g/kg), Liaoyuan (78.0 g/kg) and Urumchi (73.5 g/kg). In terms of cultivar effect, protein content varied among WPSC cultivars. The highest CP content was recorded for DK301 (85.6 g/kg) and lowest for ZD958 (74.4 g/kg). In addition, the greatest variation range of CP among different regions was found for YQ23 cultivar (69.0 to 90.9 g/kg) and the lowest for DK301 (80.6 to 91.4 g/kg).

Table 3. Crude protein (CP) chemical components and the CNCPS subfractions of whole plant silage corn cultivars grown in different regions. %

Cultivars	Regions	CP components (g/kg DM)					CNCPS subfractions (g/kg CP)				
		CP	NPN	SCP	NDICP	ADICP	PA_1	PA_2	PB_1	PB_2	PC
FKBN	*Beijing*	87.5	18.5	22.2	18.0	7.0	211.8	42.0	540.1	126.2	79.9
	Urumchi	69.5	10.2	16.6	16.5	3.7	146.6	92.7	523.6	183.3	53.9
	Cangzhou	82.1	12.6	18.9	20.1	6.1	152.9	76.6	525.7	170.3	74.6
	Tianjin	89.1	20.6	27.2	17.0	3.8	230.6	74.0	505.2	147.8	42.4
	Liaoyuan	70.7	8.90	13.9	14.6	3.5	125.7	70.5	597.4	156.8	49.6
YQ889	*Beijing*	84.8	18.7	23.8	21.3	5.7	220.7	60.1	468.6	183.7	66.9
	Urumchi	77.4	19.4	25.7	18.6	5.3	250.9	80.7	428.7	170.6	69.1
	Cangzhou	69.5	15.5	19.2	23.4	5.7	221.1	53.5	388.9	255.0	81.6
	Tianjin	80.1	8.60	16.3	28.3	5.9	106.9	96.4	443.7	279.3	73.7
	Liaoyuan	81.2	10.3	20.4	17.9	5.5	126.3	124.6	528.6	152.4	68.2
YQ23	*Beijing*	90.8	17.2	22.5	19.0	4.0	189.5	58.5	542.7	165.5	43.8
	Urumchi	69.0	12.4	19.1	13.9	3.7	179.3	97.0	521.9	148.1	53.7
	Cangzhou	84.8	13.6	17.6	29.2	5.9	159.7	47.2	448.2	275.9	69.0
	Tianjin	90.9	17.6	25.1	22.5	5.2	193.4	82.2	476.8	190.7	56.9
	Liaoyuan	82.0	11.0	17.2	21.9	5.9	133.8	76.0	522.7	196.2	71.4
DK301	*Beijing*	91.4	15.7	22.5	21.7	4.2	171.6	73.7	517.7	191.5	45.5
	Urumchi	82.6	15.0	14.2	15.5	4.0	181.5	54.6	576.2	139.9	47.8
	Cangzhou	87.9	11.6	20.0	23.1	3.9	131.2	95.1	511.2	218.4	43.9
	Tianjin	85.4	7.30	13.3	23.8	4.7	85.6	69.8	566.1	223.8	54.8
	Liaoyuan	80.6	10.6	17.2	17.6	4.5	131.2	81.8	569.4	162.1	55.6
ZD958	*Beijing*	78.5	15.9	19.1	14.8	3.1	202.6	40.5	568.8	149.1	39.0
	Urumchi	68.8	12.6	16.3	11.9	1.5	183.2	54.1	589.9	151.1	21.8
	Cangzhou	66.6	9.30	14.4	12.4	3.9	140.0	75.8	597.8	128.2	58.2
	Tianjin	82.8	13.9	19.7	14.5	3.5	167.1	69.8	589.0	131.5	42.6
	Liaoyuan	75.4	12.3	17.6	14.8	3.1	163.0	70.2	571.4	154.8	40.5

SEM		1.51	0.73	0.74	0.90	0.25	80.7	3.81	11.04	8.44	2.92
Regions											
Beijing		86.6^{a}	17.2^{a}	22.0^{a}	19.0^{b}	4.8^{ab}	199.2^{a}	55.0^{c}	527.6^{b}	163.2^{c}	55.0^{b}
Urumchi		73.5^{c}	13.9^{b}	18.4^{b}	15.3^{d}	3.6^{c}	188.3^{a}	75.8^{ab}	528.1^{b}	158.6^{c}	49.3^{c}
Cangzhou		78.2^{b}	12.5^{c}	18.0^{c}	21.6^{a}	5.1^{a}	161.0^{b}	69.6^{b}	494.4^{c}	209.6^{a}	65.5^{a}
Tianjin		85.7^{a}	13.6^{bc}	20.3^{b}	21.2^{a}	4.6^{b}	156.7^{b}	78.4^{ab}	516.2^{b}	194.6^{b}	54.1^{b}
Liaoyuan		78.0^{b}	10.6^{d}	17.3^{c}	17.4^{c}	4.5^{b}	136.0^{c}	84.6^{a}	557.9^{a}	164.5^{c}	57.1^{b}
SEM		0.55	0.43	0.41	0.40	0.012	4.56	3.43	6.37	3.58	1.30
P-value	*Region*	<.001	<.001	<.001	<.001	<.001	<.001	<.001	<.001	<.001	<.001
	Cultivar	<.001	<.001	<.001	<.001	<.001	<.001	0.002	<.001	<.001	<.001
	Region × Cultivar	<.001	<.001	<.001	<.001	<.001	<.001	<.001	<.001	<.001	<.001

Note: FKBN = Fengkenbainuo; YQ 889 = Yaqing 889; YQ 23 = Yuqing 23; DK 301 = Dongke 301; ZD958 = Zhengdan 958; 2 CP = crude protein; NPN = non-protein nitrogen; SCP = soluble crude protein; NDICP = neutral detergent insoluble crude protein; ADICP = acid detergent insoluble crude protein; 3 PA_1 = ammonia; PA_2 = soluble nonammonia CP; PB_1 = moderately degradable CP; PB_2 = slowly degradable CP; PC = unavailable CP.

Effects of growing regions and cultivars on protein *in situ* rumen degradation of whole plant silage corn

A significant interaction of corn cultivar and growing region was observed in *in situ* soluble CP (S; P = 0.002) and ruminal degradable CP (RDP; P < 0.001). However, the interactive effect was not significant (P > 0.05) in the rate of degradation (K_d), potentially ruminal degradable fraction CP (D), undegradable CP (U), and ruminal undegradable CP (RUP) (Table 4). Among *in situ* CP degradable characteristics, only the content of RUP varied (P < 0.001) among growing regions (Table 4). The highest (P < 0.05) content of RUP was observed in the WPSC grown in Beijing (41.7 g/kg DM), followed by Tianjin (39.3 g/kg DM), Liaoyuan (34.9 g/kg DM), Urumchi (34.7 g/kg DM) and Cangzhou (33.0 g/kg DM). In terms of cultivars, the highest (P < 0.05) content of RDP was observed in DK301 (47.8 g/kg DM) and lowest in ZD958 (40.2 g/kg DM). However, the highest and lowest variation of RDP content among regions was FKBN (33.7 g/kg DM in Urumchi to 53.2 g/kg DM in Tianjin) and YQ889 (38.3 g/kg DM in Cangzhou to 44.5 g/kg DM in Beijing), respectively. Cox et al. [39] reported that *in vitro* NDF digestibility of six corn hybrids on average varied from 710 to 827 g/kg between two growing regions, which indirectly explained our results. But limited information exists to make a comparison of *in situ* CP rumen degradation kinetics for forages cultivated in different regions. The variation of RDP in WPSC from different regions was highest in FKBN (39.3 to 53.2 g/kg DM), followed by YQ23 (38.3 to 46.5 g/kg DM), DK301 (43.5 to 47.9 g/kg DM), ZD958 (36.0 to 44.9 g/kg DM) and YQ889 (39.8-44.5 g/kg DM). The result was not fully consistent with the variation range observed in CP content.

Table 4. In situ crude protein (CP) degradation kinetics of whole plant silage corn cultivars grown in different regions. %

Cultivars	Regions	K_d (/h)	S (g/kg CP)	D (g/kg CP)	RUP	RDP (g/kg DM)

					(g/kg DM)	
FKBN	*Beijing*	0.080	479.8	520.2	40.20	47.30
	Urumchi	0.021	371.4	465.3	35.79	33.71
	Cangzhou	0.029	381.5	472.0	38.81	43.29
	Tianjin	0.026	536.9	206.3	35.89	53.21
	Liaoyuan	0.020	413.3	586.7	31.37	39.33
YQ889	*Beijing*	0.022	464.4	281.4	40.26	44.54
	Urumchi	0.067	422.0	193.1	37.55	39.85
	Cangzhou	0.029	456.0	311.0	31.21	38.29
	Tianjin	0.006	443.3	556.7	40.35	39.75
	Liaoyuan	0.012	455.7	544.3	36.74	44.46
YQ23	*Beijing*	0.015	431.8	454.6	44.27	46.53
	Urumchi	0.013	484.6	422.6	30.73	38.27
	Cangzhou	0.049	501.6	282.9	31.51	53.29
	Tianjin	0.023	441.9	346.7	44.68	46.22
	Liaoyuan	0.029	428.2	401.7	38.74	43.26
DK301	*Beijing*	0.012	373.4	626.6	47.92	43.48
	Urumchi	0.013	463.4	509.7	36.82	45.78
	Cangzhou	0.063	409.4	396.4	34.15	53.75
	Tianjin	0.171	367.2	417.6	37.55	47.85
	Liaoyuan	0.016	489.3	510.7	32.72	47.88
ZD958	*Beijing*	0.012	45.30	52.79	36.01	42.49
	Urumchi	0.017	39.45	60.55	32.80	36.00
	Cangzhou	0.012	46.73	53.27	29.45	37.15
	Tianjin	0.150	42.91	34.85	37.88	44.92
	Liaoyuan	0.015	41.49	58.51	35.11	40.29
	SEM	0.0718	0.845	2.422	0.905	1.040
Regions						
	Beijing	0.014^{b}	44.05	48.21^{ab}	41.73^{a}	44.87^{ab}
	Urumchi	0.026^{ab}	42.72	43.92^{ab}	34.74^{b}	38.72^{c}
	Cangzhou	0.036^{ab}	44.32	39.90^{ab}	33.03^{b}	45.15^{ab}

Tianjin		0.066[a]	44.37	37.52[b]	39.27[a]	46.39[a]
Liaoyuan		0.019[b]	44.03	52.57[a]	34.94[b]	43.04[b]
SEM		0.0157	1.158	4.550	1.127	1.127
P-value	*Region*	0.016	0.85	0.016	<0.001	<0.001
	Cultivar	0.59	0.22	0.13	0.16	<0.001
	Region × Cultivar	0.48	0.002	0.29	0.06	<0.001

Note: FKBN = Fengkenbainuo; YQ 889 = Yaqing 889; YQ 23 = Yuqing 23; DK 301 = Dongke 301; ZD958 = Zhengdan 958; 2 K_d = ruminal degradation rate; S = soluble CP in the *in situ* incubation; D = insoluble but potentially degradable CP in the *in situ* incubation; U = potential undegradable CP in the *in situ* incubation; RUP = the crude protein fraction of bypass rumen; EDCP = the effective degradable fraction of crude protein in the rumen.

Effects of growing regions and cultivars on protein molecular structures of whole plant silage corn

Previous studies showed that feed protein solubility, ruminal degradability and post-ruminal digestibility not only depended on CP chemical compositions, but also strongly influenced by protein inherent molecular structures [40-42]. Therefore, differences in protein spectral characteristics of WPSC were quantified and visualized between growing regions and corn cultivars.

Data on secondary protein structures showed an interactive effect of growing region and cultivar for *α*-helix and *β*-sheet height (Table 5, $P < 0.001$). However, no significant interaction was observed on the *α*-helix to *β*-sheet height ratio ($P = 0.26$). The ration of *α*-helix to *β*-sheet height varied ($P < 0.001$) among growing regions, specifically, the highest ratio was recorded for Urumchi (1.88) and lowest for Beijing (1.02). In agreement with our findings, previous study reported that crops under water stress exhibited significantly different CP structural features and carbohydrates spectral regions compared to crops grown in dry land [6, 8]. During the growing and development period, the plants need to absorb and accumulate nutrients by the roots from soil, and water is the key transmission medium for transportation of all nutrients. Therefore, sufficient soil water could contribute to high efficiency of nutrients transportation and promotion of cell elongation [43], which might further cause a great development of molecular makeup and conformation of biopolymers in plants. The highest variation in protein amide area across the growing regions was observed in YQ889 (2.5 to 11.7 units) and lowest in DK301 (5.4 to 9.0 units).

The results of CLA and PCA analyses were presented in Figure 1. It was evident from Figure 1 that the protein structural makeup of FKBN and DK301 cultivated in Tianjin were distinguished from those grown in other regions. As for cultivars of YQ889 and YQ23, clear separate ellipses were found between Cangzhou and other growing locations. For the remaining cultivars, heavy overlaps were observed for the protein primary structural data. These results indicated that for some WPSC cultivars, internal protein molecular makeups were altered when environmental conditions changed, which was partly consistent with our data from univariate spectral analysis (Table 5).

Table 5. Protein molecular structures of silage corn cultivars grown in different regions. **%**

Cultivars	Regions	Protein Primary Structure Characteristics	Protein Secondary Structure

		Amide peak I height	Amide peak II height	Amide peak (I: II) height ratio	Amide peak I area	Amide peak II area	Amide peak (I: II) area ratio	α-helix height	β-sheet height	Height ratio α- helix: β-sheet
FKBN	*Beijing*	0.07	0.01	5.34	4.79	0.61	7.87	0.07	0.06	1.20
	Urumchi	0.05	0.01	3.27	3.71	0.36	10.28	0.08	0.04	2.40
	Cangzhou	0.07	0.02	3.68	5.32	0.52	10.83	0.07	0.07	0.98
	Tianjin	0.06	0.01	4.30	5.20	0.39	13.44	0.07	0.06	1.16
	Liaoyuan	0.13	0.03	4.72	10.2	0.69	11.47	0.12	0.13	0.98
YQ889	*Beijing*	0.05	0.01	4.45	3.84	0.33	11.63	0.05	0.04	1.38
	Urumchi	0.14	0.04	3.13	10.72	1.17	9.20	0.14	0.07	1.97
	Cangzhou	0.10	0.02	5.83	6.51	0.78	8.35	0.09	0.08	1.25
	Tianjin	0.03	0.005	6.01	2.41	0.22	11.1	0.03	0.03	1.03
	Liaoyuan	0.11	0.02	4.51	7.99	0.71	11.29	0.10	0.10	1.01
YQ23	*Beijing*	0.03	0.002	20.17	2.58	0.26	9.97	0.03	0.03	0.85
	Urumchi	0.08	0.02	4.72	5.96	0.61	9.91	0.08	0.06	1.54
	Cangzhou	0.07	0.02	3.78	5.11	0.52	9.89	0.07	0.04	1.60
	Tianjin	0.11	0.03	4.09	8.38	0.82	10.09	0.10	0.11	0.92
	Liaoyuan	0.09	0.02	4.83	6.67	0.67	9.92	0.09	0.08	1.04
DK301	*Beijing*	0.12	0.004	28.83	8.84	0.97	9.09	0.08	0.12	0.69
	Urumchi	0.07	0.01	4.76	5.55	0.59	9.21	0.08	0.07	1.20
	Cangzhou	0.07	0.02	4.01	4.78	0.55	8.67	0.06	0.06	1.15
	Tianjin	0.11	0.02	6.06	7.84	0.58	13.91	0.10	0.10	1.03
	Liaoyuan	0.07	0.02	3.99	5.2	0.65	8.04	0.07	0.06	1.20
ZD958	*Beijing*	0.10	0.01	10.31	7.68	0.68	11.33	0.10	0.10	0.99
	Urumchi	0.09	0.03	3.51	7.47	0.77	9.69	0.09	0.04	2.29
	Cangzhou	0.13	0.03	5.05	9.36	0.84	12.2	0.12	0.13	0.97
	Tianjin	0.10	0.02	4.15	6.91	0.82	8.30	0.09	0.08	1.20
	Liaoyuan	0.10	0.02	5.65	7.17	0.47	15.35	0.09	0.09	0.98
	SEM	0.006	0.002	1.131	0.431	0.043	0.366	0.005	0.006	0.083
Regions										
	Beijing	0.07^{b}	0.008^{c}	13.82^{a}	5.55^{b}	0.57	9.98	0.063^{b}	0.069^{bc}	1.02^{b}

Urumchi		0.09^{ab}	0.023^{a}	3.88^{b}	6.68^{ab}	0.70	9.66	0.093^{a}	0.055^{c}	1.88^{a}
Cangzhou		0.09^{ab}	0.019^{ab}	4.47^{b}	6.22^{ab}	0.64	9.99	0.083^{a}	0.074^{b}	1.19^{b}
Tianjin		0.08^{b}	0.017^{b}	4.92^{b}	6.15^{b}	0.57	11.37	0.078^{ab}	0.075^{b}	1.07^{b}
Liaoyuan		0.10^{a}	0.021^{a}	4.74^{b}	7.45^{a}	0.64	17.81	0.094^{a}	0.092^{a}	1.04^{b}
SEM		0.006	0.001	0.857	0.438	0.050	2.974	0.006	0.006	0.117
P-value	*Region*	0.048	<.001	<.001	0.047	0.28	0.02	0.005	<.001	<.001
	Cultivar	0.01	0.01	<.001	0.02	0.053	0.08	0.050	0.04	0.39
	Region × Cultivar	<.001	<.001	<.001	<.001	<.001	<.001	<.001	<.001	0.26

Note: FKBN = Fengkenbainuo; YQ 889 = Yaqing 889; YQ 23 = Yuqing 23; DK 301 = Dongke 301; ZD958 = Zhengdan 958.

Correlation of protein molecular structural features with protein chemical profiles as well as in situ degradation parameters of whole plant silage corn

Figure 2 visualized results of the relationship between protein molecular structural features and CP chemical profiles, CNCPS subfractions and *in situ* rumen degradation characteristics. The area ratio of amide I to II was negatively correlated with the contents of SCP (δ = -0.66; *P* = 0.002), CP (δ = -0.61; *P* = 0.006), NPN (δ = -0.56; *P* = 0.004), ADICP (δ = -0.43; *P* = 0.008) and PA_1(δ = -0.38; *P* = 0.047), whereas positively correlated with PB_1 (δ = 0.58; *P* = 0.01). The *α*-helix to *β*-sheet height ratio was positively correlated with PB_2 (δ = 0.22; *P* = 0.048) while negatively correlated with PB_1 (δ = -0.46; *P* = 0.002). Similarly, the correlation between protein molecular structures and chemical profiles was demonstrated by previous study [44]. However, Liu et al. [45] found negative relationships between *α*-helix to *β*-sheet height ratio with PB_2 (δ = -0.45; $P < 0.01$) in wheat, corn and triticale grains, which were not in line with current study.

The amide I to II area ratio had a negative correlation with RUP content (δ = -0.48; *P* = 0.02). The relations of spectral characteristics with *in situ* CP ruminal degradation kinetics were not significant. Numerous studies have reported correlations between protein molecular structural traits and ruminal degradation parameters in varieties of feedstuff [46-47]. For instance, Zhang and Yu [46] represented relationships of amide I area (δ =0.97; *P* = 0.005), amide II area (δ = 0.90; *P* = 0.036), *α*-helix height (δ = 0.96; *P* = 0.011), *β*-sheet height (δ = 0.98; *P* = 0.004), in conjunction with *α*-helix to *β*-sheet height ratio (δ = -0.99; *P* = 0.00) and RDP in hulless barley and bioethanol coproducts of wheat or dried distillers grains with solubles. However, Gomaa et al. [36] suggested that no relationship was observed between area ratio of amide I to II and RUP in canola seeds. Although the data was very limited, these inconsistent results showed that the correlations between protein molecular structural characteristics and CP chemical profiles as well as rumen degradable characteristics were specific to some extent.

FKBN

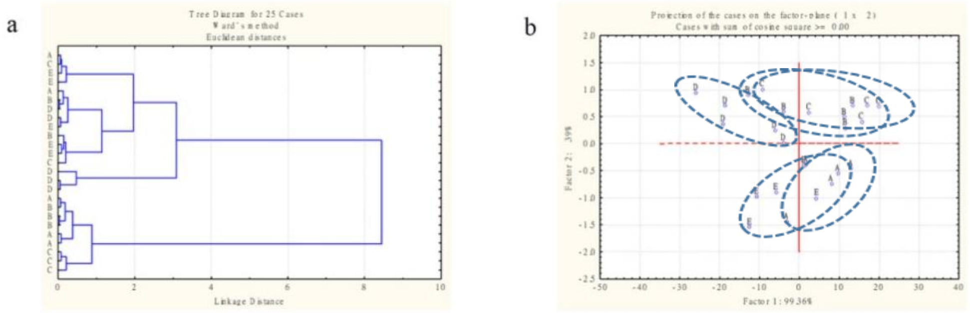

YQ889

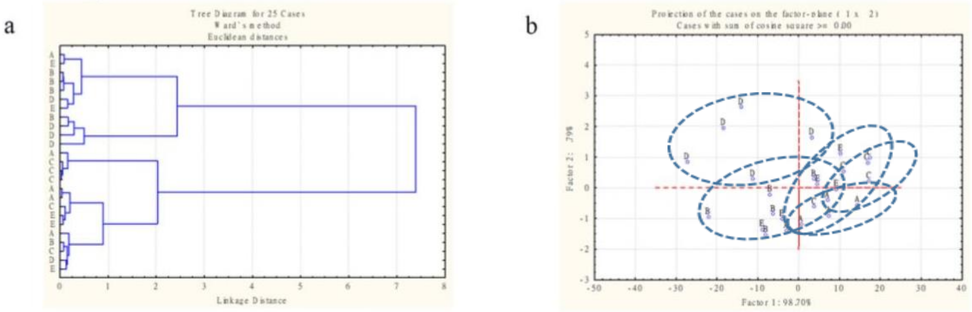

YQ23

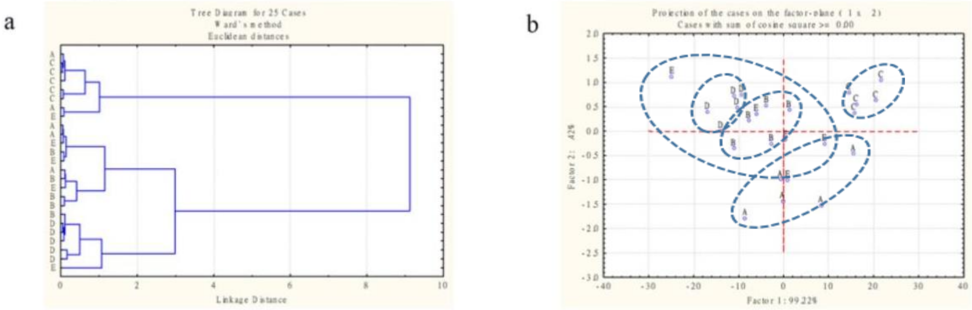

DK301

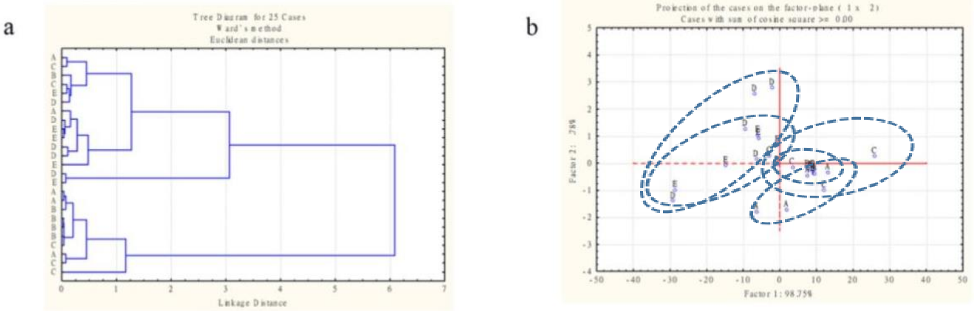

ZD958

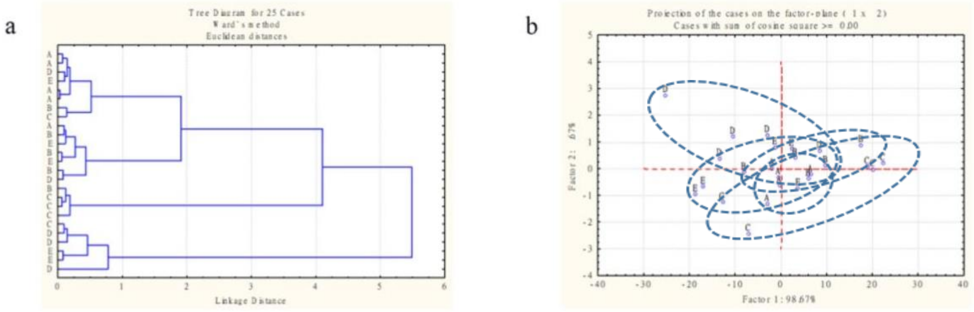

Fig. 1. Multivariate molecular spectral analyses of protein molecular spectral feature in protein amide I and amide II fingerprint region (ca. 1712–1489 cm^{-1}) of various whole-plant silage corn cultivars from different regions. a: CLA analysis; b: PCA analysis; A = Beijing; B = Urumchi; C = Cangzhou; D = Tianjin; E = Liaoyuan

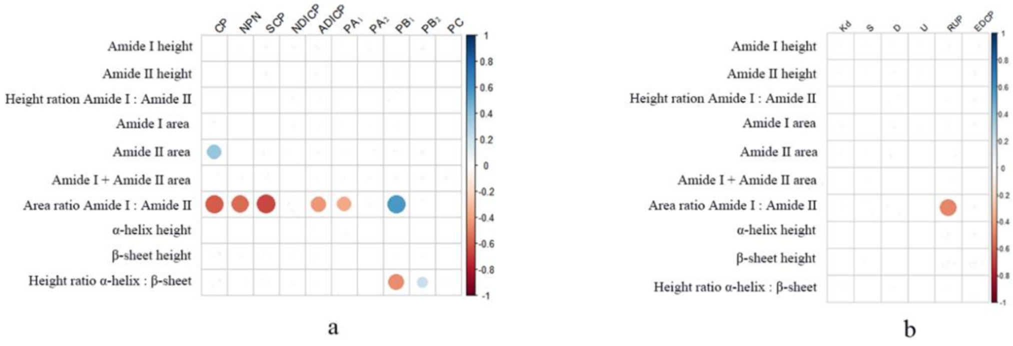

Fig. 2. Correlation of protein molecular structural features with chemical profile and CNCPS subfractions and in situ biodegradation parameters. a, correlation of protein molecular structure features with protein chemical profile and CNCPS subfractions; b, correlation of protein molecular structure features with in situ biodegradation parameters. CP, crude protein; NPN, non-protein nitrogen; SCP, soluble CP; NDICP, neutral detergent insoluble CP; ADICP, acid detergent insoluble CP; PA_1, ammonia; PA_2, soluble non-ammonia CP; PB_1, moderately degradable CP; PB_2, slowly degradable CP; PC, unavailable CP; K_d, ruminal degradation rate; S, soluble CP in the in situ incubation; D, insoluble but potentially degradable CP in the in situ incubation; U, potential undegradable CP in the in situ incubation; RUP, the CP fraction of bypass rumen; EDCP, the effective degradable fraction of CP in the rumen.

Conclusion

In conclusion, there were significant interaction between growing region and WPSC cultivar for most of the measured morphological characteristics, CP chemical profiles, CNCPS subfractions, *in situ* ruminal degradation parameters, and protein molecular structural features. In detail, our study showed that even for the same cultivar, the protein nutritional values and molecular structures significantly varied between growing regions. Besides, the between-region variations in protein chemical profiles, CNCPS subfractions and *in situ* degradation parameters were strongly correlated with some protein molecular spectral parameters. Further in-depth studies are required to establish some predictive models based on novel technology combined with reliable algorithm for estimating nutritional contents and rumen degradable characteristics.

Acknowledgments

The authors would like to thank supplementation from Northeast Agricultural University.

References

1. Khaing KT, Loh TC, Ghizan S, Halim RA, Samsudin AA. Feed intake, growth performance and digestibility in goats fed whole corn plant silage and Napier grass. Mal J Ani Sci.2015 Jun;18(1):87-97.
2. Khan NA, Yu P, Ali M, Cone JW, Hendriks WH. Nutritive value of maize silage in relation to dairy cow performance and milk quality. J Sci Food Agric. 2015 Jan;95(2):238-52.
3. Khan NA, Tewoldebrhan TA, Zom RL, Cone JW, Hendriks WH. Effect of corn silage harvest maturity and concentrate type on milk fatty acid composition of dairy cows. J Dairy Sci. 2012 Mar;95(3):1472-83.
4. Khan NA, Peng Q, Xin H, Yu P. Vibrational spectroscopic investigation of heat-induced

changes in functional groups related to protein structural conformation in camelina seeds and their relationship to digestion in dairy cows. Anim Pro Sci. 2015 Aug;55(2):201–206.
5. Ferraretto LF, Shaver RD. Effects of whole-plant corn silage hybrid type on intake, digestion, ruminal fermentation, and lactation performance by dairy cows through a meta-analysis. J Dairy Sci. 2015 Apr;98(4):2662-75.
6. Abeysekara S, Khan NA, Yu P. Relationship between protein molecular structural makeup and metabolizable protein supply to dairy cattle from new cool-season forage corn cultivars. Spectrochim Acta A Mol Biomol Spectrosc. 2018 Feb 15;191:303-314.
7. Bernardes TF, Daniel JLP, Adesogan AT, McAllister TA, Drouin P, Nussio LG, Huhtanen P, Tremblay GF, Bélanger G, Cai Y. Silage review: Unique challenges of silages made in hot and cold regions. J Dairy Sci. 2018 May;101(5):4001-4019.
8. Kung L Jr, Shaver RD, Grant RJ, Schmidt RJ. Silage review: Interpretation of chemical, microbial, and organoleptic components of silages. J Dairy Sci. 2018 May;101(5):4020-4033.
9. Temime SB, Campeol E, Cioni PL, Daoud D, Zarrouk M. Volatile compounds from Chétoui olive oil and variations induced by growing area. Food Chem. 2006 Jul;99(2):315-325.
10. Rengsirikul K, Ishii Y, Kangvansaichol K, Sripichitt P, Punsuvon V, Vaithanomsat P, Nakamanee, G., Tudsri, S. Biomass yield, chemical composition and potential ethanol yields of 8 cultivars of napiergrass (Pennisetum purpureum Schumach.) harvested 3-monthly in central Thailand. J Sustain Bioenergy Syst. 2013 Jun;3(2):107-112.
11. Hao JY, Wan Y, Yao XH, Zhao WG, Hu RZ, Chen C, Li L, Zhang DY, Wu GH. Effect of different planting areas on the chemical compositions and hypoglycemic and antioxidant activities of mulberry leaf extracts in Southern China. PLoS One. 2018 Jun 26;13(6):e0198072.
12. Peng Q, Khan NA, Wang Z, Zhang X, Yu P. Effect of thermal processing on estimated metabolizable protein supply to dairy cattle from camelina seeds: relationship with protein molecular structural changes. J Agric Food Chem. 2014 Aug 20;62(33):8263-73.
13. Zhang X, Yu P. Relationship of carbohydrate molecular spectroscopic features in combined feeds to carbohydrate utilization and availability in ruminants. Spectrochim Acta A Mol Biomol Spectrosc. 2012 Jun 15;92:225-33.
14. Yu P. Protein secondary structures (alpha-helix and beta-sheet) at a cellular level and protein fractions in relation to rumen degradation behaviours of protein: a new approach. Br J Nutr. 2005 Nov;94(5):655-65.
15. Yu P. Protein Molecular Structures, Protein Subfractions and Protein Availability Affected by Heat Processing: A Review. Am J Biochem Biotechnol. 2007 Jan;3(2):66–86.
16. Becker PM, Yu P. What makes protein indigestible from tissue-related, cellular, and molecular aspects? Mol Nutr Food Res. 2013 Oct;57(10):1695-707.
17. Doiron K, Yu P, McKinnon JJ, Christensen DA. Heat-induced protein structure and subfractions in relation to protein degradation kinetics and intestinal availability in dairy cattle. J Dairy Sci. 2009 Jul;92(7):3319-30.
18. Yu P, Prates LL. Molecular structure of feeds in relation to nutrient utilization and availability in animals: a novel approach. Engineering. 2017 Oct;3(5):726–730.
19. Sun B, Khan NA, Yu P. Molecular spectroscopic features of protein in newly developed chickpea: Relationship with protein chemical profile and metabolism in the rumen and intestine of dairy cows. Spectrochim Acta A Mol Biomol Spectrosc. 2018 May 5;196:168-177.
20. AOAC. Association of Official Analytical Chemists, Official methods of analysis, 17th ed. 2000. AOAC, Arlington, VA, USA.
21. Licitra G, Hernandez TM, Van Soest PJ. Standardization of procedures for nitrogen fractionation of ruminant feeds. Anim Feed Sci Technol. 1996 Mar;57(4):347-358
22. Higgs RJ, Chase LE, Ross DA, Van Amburgh ME. Updating the Cornell Net Carbohydrate and Protein System feed library and analyzing model sensitivity to feed inputs. J Dairy Sci. 2015 Sep;98(9):6340-60.
23. Van Amburgh ME, Foskolos A, Collao-Saenz E, Higgs R, Ross DA. Updating the CNCPS feed

library with new feed amino acid profiles and efficiencies of use: Evaluation of model predictions, version 6.5. 2013. 75th Cornell Nutrition Conference for Feed Manufacturers.
24. Ørskov ER, McDonald I. The estimation of protein degradability in the rumen from incubation measurements weighted according to rate of passage. J Agric Sci. 1979 Mar;92:499-503.
25. Yu P. Applications of hierarchical cluster analysis (CLA) and principal component analysis (PCA) in feed structure and feed molecular chemistry research, using synchrotron-based Fourier transform infrared (FTIR) microspectroscopy. J Agric Food Chem. 2005 Sep 7;53(18):7115-27.
26. Huxman TE, Smith MD, Fay PA, Knapp AK, Shaw MR, Loik ME, Smith SD, Tissue DT, Zak JC, Weltzin JF, Pockman WT, Sala OE, Haddad BM, Harte J, Koch GW, Schwinning S, Small EE, Williams DG. Convergence across biomes to a common rain-use efficiency. Nature. 2004 Jun 10;429(6992):651-4.
27. Polania JA, Salazar-Chavarría V, Gonzalez-Lemes I, Acosta-Maspons A, Chater CCC, Covarrubias AA. Contrasting PhaseolusCrop Water Use Patterns and Stomatal Dynamics in Response to Terminal Drought. Front Plant Sci. 2022 May 31;13:894657.
28. Habermann E, San Martin JAB, Contin DR, Bossan VP, Barboza A, Braga MR, Groppo M, Martinez CA. Increasing atmospheric CO2 and canopy temperature induces anatomical and physiological changes in leaves of the C4 forage species Panicum maximum. PLoS One. 2019 Feb 19;14(2):e0212506.
29. Wang N, Wang E, Wang J, Jiang JP. Modelling maize phenology, biomass growth and yield under contrasting temperature conditions. Agric Meteorol. 2018 Mar;s250–251:319-329.
30. Shi D, Huang Q, Liu Z, Liu T, Su Z, Guo S, Bai F, Sun S, Lin X, Li T, Yang X. Radiation use efficiency and biomass production of maize under optimal growth conditions in Northeast China. Sci Total Environ. 2022 Aug 25;836:155574.
31. Tollenaar M, Fridgen J, Tyagi P, Stackhouse PW Jr, Kumudini S. The Contribution of Solar Brightening to the US Maize Yield Trend. Nat Clim Chang. 2017 Mar 6;7:275-278..
32. Andrade FH, Otegui ME, Vega C. Intercepted radiation at flowering and kernel number in maize. Semi Forum. 2000 Jan;92(1):92–97
33. Russell JB, O'Connor JD, Fox DG, Van Soest PJ, Sniffen CJ. A net carbohydrate and protein system for evaluating cattle diets: I. Ruminal fermentation. J Anim Sci. 1992 Nov;70(11):3551-61.
34. Fox DG, Sniffen CJ, O'Connor JD, Russell JB, Van Soest PJ. A net carbohydrate and protein system for evaluating cattle diets: III. Cattle requirements and diet adequacy. J Anim Sci. 1992 Nov;70(11):3578-96.
35. O'Connor JD, Sniffen CJ, Fox DG, Chalupa W. A net carbohydrate and protein system for evaluating cattle diets: IV. Predicting amino acid adequacy. J Anim Sci. 1993 May;71(5):1298-311.
36. Gomaa WMS, Peng Q, Mosaad GM, Zhang H, Yu P. Protein molecular structure in relation to predicted biodegradation and nutrient supply of feedstocks and co-products from bio-oil processing with CNCPS system: comparison crusher plants within Canada and within China as well as between Canada and China. Anim Feed Sci Technol. 2018 Sep;243:125-139.
37. Gyuricza C, Balla I, Tarnawa Á, Nyárai FH, Kassai K, Szentpétery Z, Jolánkai M. Impact of precipitation on yield quantity and quality of wheat and maize crops. J Hung Meteor Service. 2012 Jul;116(3):211-220.
38. Cone JW, Engels FM. Influence of growth temperature on anatomy and in vitro digestibility of maize tissue. J Agric Sci. 1990 Mar;114:207-212.
39. Cox WJ, Cherney JH, Pardee WD, Cherney DJR. Forage quality and harvest index of corn hybrids under different growing conditions. Agron J. 1994 Mar;86(2):277-282.
40. Yu P. Synchrotron IR microspectroscopy for protein structure analysis: potential and questions. Spectrosc. 2006 Jan;20(5-6):229-251.
41. Yu P, Jonker A, Gruber M. Molecular basis of protein structure in proanthocyanidin and

anthocyanin-enhanced Lc-transgenic alfalfa in relation to nutritive value using synchrotron-radiation FTIR microspectroscopy: a novel approach. Spectrochim Acta A Mol Biomol Spectrosc. 2009 Sep 1;73(5):846-53.
42. Yu P, Nuez-Ortín WG. Relationship of protein molecular structure to metabolisable proteins in different types of dried distillers grains with solubles: a novel approach. Br J Nutr. 2010 Nov;104(10):1429-37.
43. Scharwies JD, Dinneny JR. Water transport, perception, and response in plants. J Plant Res. 2019 May;132(3):311-324.
44. Ban Y. Molecular structural, physiochemical and nutritional characterization of new lines of Brassica carinata and the co-products. University of Saskatchewan, Saskatoon, SA. 2016.
45. Liu B, Thacker P, McKinnon J, Yu P. In-depth study of the protein molecular structures of different types of dried distillers grains with solubles and their relationship to digestive characteristics. J Sci Food Agric. 2013 Apr;93(6):1438-48.
46. Zhang X, Yu P. Molecular basis of protein structure in combined feeds (hulless barley with bioethanol coproduct of wheat dried distillers grains with solubles) in relation to protein rumen degradation kinetics and intestinal availability in dairy cattle. J Dairy Sci. 2012 Jun;95(6):3363-79.
47. Li X, Zhang Y, Yu P. Association of Bio-energy Processing-Induced Protein Molecular Structure Changes with CNCPS-Based Protein Degradation and Digestion of Co-products in Dairy Cows. J Agric Food Chem. 2016 May 25;64(20):4086-94.
