# Supplementary material for: Effect of growing regions on morphological characteristics, protein subfractions, rumen degradation and molecular structures of various whole-plant silage corn cultivars
Source: PLoS One. 2024 Jan 11;19(1):e0282547. doi: 10.1371/journal.pone.0282547 (PMC10783724; doi:10.1371/journal.pone.0282547)
Supplement: Attachment — Submitted filename: Response to Reviewers.docx [file pone.0282547.s004.docx]

Reviewer #1:

Lack of novelty, originality and in vivo experiments to the existing study of the field as well as regional research work in order to add to the existing knowledge unless the methodology of the work done demonstrates something new.

Response:

Thanks for the review’s comments. We apologize for the ambiguous intent in this manuscript, and the integrated logistics about this research was reorganized as follow. We sincerely hope that our logic now is clearer to exhibit novelty and originality.

On the one hand, although previous studies have demonstrated that the different nutrient components between various forages, it was not comprehensive and lack representativeness. In current study, our sample collections and detected indicators were comprehensive, which could offset the problem involved in previous studies.

On the other hand, the Chinese dairy industry has developed rapidly over recent decades and needs to provide milk products to a quarter of the world's population. This further requires sustainable supplementation of feedstuffs to adapt the intensive systems. It is important to assess the nutritional concentrations and rumen degradation of forage before it can be used in the dairy industry. However, the traditional laboratory analysis for roughage costs a mass of human, material, and financial resources, as well contributes to some errors due to different operators and instruments. Therefore, as a rapid, accurate, and portable technology, Fourier transform infrared (FTIR) spectroscopy could be applied to establish a model for predicting nutrient components and digestibility. However, to our knowledge, the whole plant silage corn samples utilized to build models was only limited in a specific site. I remember that a predictive model for nutrient components of forage established by my senior sister, who collected samples only in Beijing. So, we next plan to construct a predictive model based on the data collected across the country. Above all, this manuscript represented the first section of our work. That is the necessary to build efficient predictable model to estimate nutrients of whole plant silage corn before they were utilized because of huge variation induced by cultivars and grown regions.

Anyway, sorry for the confusion brought the reviewer again, and we really hope this explanation will help the reviewer understand this manuscript better.

Reviewer #2:

The manuscript evaluates the variation of morphological characteristics, nutritional value, ruminal degradability, and molecular structural makeup of diverse whole-plant silage corn cultivars among different growing regions. The study suggested that the morphological characteristics, protein nutritional values and rumen degradability significantly varied among different grown regions due to distinguished molecular structures. The manuscript is novel and important for the readers. However, some inquiries need to be clarified before the final decision.
(1) The English in the text should be improved.
(2) My main concern is the statistical analysis. Please explain in details the CLA and PCA analysis. Could you send me the code used for this analysis? what was your experimental unit for all of your obtained results? which significance degree did you use?
(3) Please insert more information about the plant collection, plant sample size? how many trees were collected for each plant sample? how many plots are for each plant sample? how many true statistical and analytical repetitions were made for all the obtained results?
(4) There may be need to know how much feed the animals were given to ensure maintenance energy intake. The same applies to the amount of treatment samples incubated for degradation, no details on the amount of samples in terms of weight per bag and the number of bags per animal.

Response:

The statements have been corrected. We will be happy to edit the text further based on the helpful comments from the review.

(1) We apologize for the poor language of our manuscript. We worked on the manuscript for a long time and the sentences obviously led to poor readability were revised repeatedly or removed. Now, we really hope that the flow and language level have been substantially improved.
(2) Sorry for the confusion. There was no code, because the Statistica 8.0 software was used to analyze the CLA and PCA. For the cluster analyses, Ward’s Algorithm method was used for clustering calculation, with results displayed as dendrograms (Yu, 2010), and the Euclidean method was applied to the distance matrix calculation. In the Principal Component Analysis, all original variables were transferred to a set of new uncorrelated variables called principal components (PCs). Then, the first principal component (PC1) and second principal component (PC2) were generated in a scatter plot and used to describe all variables. The specific spectral analyses of these two methods were explained in Yu (2005, 2010).

Reference:

Yu P. Applications of hierarchical cluster analysis (CLA) and principal component analysis (PCA) in feed structure and feed molecular chemistry research, using synchrotron-149 based Fourier transform infrared (FTIR) microspectroscopy. J Agric Food Chem. 2005 Sep; 53:7115-7127.

Yu P. Plant-based food and feed protein structure changes induced by gene-transformation, heating and bio-ethanol processing: a synchrotron-based molecular structure and nutrition research program. Mol Nutr Food Res. 2010 Nov; 54:1535-1545.

(3) Total five cultivars of WPCS (FKBN, YQ889, YQ23, DK301 and ZD958) were selected for this study from five different areas (Beijing, Urumchi, Cangzhou, Liaoyuan, Tianjin) of China. In each area, three plots were selected, and 20 plants from each plot, 10 cm above the ground, were randomly harvested at the kernel maturity stage of the half milk line. The exterior 1 m area of each plot was excluded from sampling to ensure uniformity in the plants being sampled. Anyway, from the point of statistic, the average value of nutrient indicators from 20 WPCS plants were seemed as raw data, three plots of per sample were treated as replicates. Sorry for the unclear description in original manuscript, we have corrected it and really hope that the new version could be more accurate.

(4) Sorry for the careless, the ingredients and nutrient contents of basal diet for dairy cows were added in this manuscript (Table 2). Each nylon bag (10 × 20 cm, pore size 40 μm) was placed 7 g WPSC, and three replicates were set for per sample at every incubation time point. The “gradual in/all out” schedule was used in this experiment, so the number of bags were changed at different time point, but the total number was no more than 30 nylon bags for each animal. Specifically, a total number of 75 nylon bags (25 samples × 3 replicates) at each time point were randomly selected for three individual dairy cows, and the nutrient degradation process was conducted twice as the random effect of statistical analysis in this trial. The detailed content was added in the material and methods section of this manuscript.

Reviewer #3:

It feels like a record of in-house experimentation at a seed company.
Isn't it natural to expect that plants with different genetic traits, grown in different climates, will show different growth processes?
I don't think this is a new scientific law.
Did you get permission from the ethics committee to conduct animal experiments? What is the permission number?
Other minor comments were redacted in the manuscript.

Response:

Thanks for the review’s comments. We apologize for the ambiguous intent in this manuscript, and the integrated logistics about this research was reorganized as follow. We sincerely hope that our logic now is clearer to exhibit novelty and originality.

On the one hand, although previous studies have demonstrated that the different nutrient components between various forages, it was not comprehensive and lack representativeness. In current study, our sample collections and detected indicators were comprehensive, which could offset the problem involved in previous studies.

On the other hand, the Chinese dairy industry has developed rapidly over recent decades and needs to provide milk products to a quarter of the world's population. This further requires sustainable supplementation of feedstuffs to adapt the intensive systems. It is important to assess the nutritional concentrations and rumen degradation of forage before it can be used in the dairy industry. However, the traditional laboratory analysis for roughage costs a mass of human, material, and financial resources, as well contributes to some errors due to different operators and instruments. Therefore, as a rapid, accurate, and portable technology, Fourier transform infrared (FTIR) spectroscopy could be applied to establish a model for predicting nutrient components and digestibility. However, to our knowledge, the whole plant silage corn samples utilized to build models was only limited in a specific site. I remember that a predictive model for nutrient components of forage established by my senior sister, who collected samples only in Beijing. So, we next plan to construct a predictive model based on the data collected across the country. Above all, this manuscript represented the first section of our work. That is the necessary to build efficient predictable model to estimate nutrients of whole plant silage corn before they were utilized because of huge variation induced by cultivars and grown regions.

Anyway, sorry for the confusion brought the reviewer again, and we really hope this explanation will help the reviewer understand this manuscript better.

As for the ethics committee, our study was approved by the Ethical Committee of the College of Animal Science and Technology, Northeast Agriculture University for animal experiment (NEAU-[2011]-9).

Finally, the statements noted in this manuscript were corrected seriously step by step. We will be happy to edit the text further based on the helpful comments from the review.

Reviewer #4:

The topic of the manuscript is interesting and includes practical findings. However, it has issues that need to be fixed. In particular, the Materials and Methods, Discussion and Conclusion sections should be improved. Please see the annotated file for some comments.

Response:

Thanks for the review’s suggestions, and we have carefully revised the relative contents in accordance with the comments.

Reviewer #5:

1.statistic difficult to understand
2.Treatment should classified more difficult to understand
3.This manuscript how many experiment difficult to understand
4.Should recheck format of journal
5.Should recheck grammar or ask English personal to proof

Response:

Thanks for the review’s suggestions. According to his/her advice, we have reorganized the inappropriate contents in the new version of the manuscript. We will be happy to edit the text further based on the helpful comments from the review.
