# Supplementary material for: Effect of growing regions on morphological characteristics, protein subfractions, rumen degradation and molecular structures of various whole-plant silage corn cultivars
Source: PLoS One. 2024 Jan 11;19(1):e0282547. doi: 10.1371/journal.pone.0282547 (PMC10783724; doi:10.1371/journal.pone.0282547)
Supplement: Attachment — Submitted filename: Response to Reviewers.docx [file pone.0282547.s005.docx]

**Additional Editor Comments:**
1) The manuscript contains many grammatical, typographic, and styling errors. English editing of the manuscript by a native English speaker is highly recommended.
2) The research question is not clearly outlined in the Introduction section.
3) Figure 1 has a low quality and should be presented in higher resolution.
4) The discussion is superficial. All estimated parameters should be discussed in more detail with more depth. Also, the discussion must be fortified with possible attributions.

**Response:**

Thanks for your suggestions and we have carefully revised the relative contents in accordance with the comments.

Firstly, we rechecked the manuscript by a native English speaker at your suggestion.

Then, the introduction and discussion sections were revised appropriately. For introduction part, we give a brief introduction of whole-plant silage corn (WPSC) and its important role in first paragraph, and the environmental factors for WPSC growth were discussed in second paragraph. Then, the association of protein chemical profiles and *in situ* degradation parameters with the spectral data of specific inherent structures were analyzed. In terms of discussion part, some new references were inserted in the manuscript to discuss more depth.

Finally, a clearer Figure 1 was reuploaded.

Anyway, thank you again, and we all be happy to edit the text further based on the helpful comments from you!

**Reviewer #4:**

The manuscript has been partially improved. Some things still need to be corrected and clarified.

In the section “Corn cultivars, growing regions, and crop production”:
More clarification is needed about the practical management and agronomic practices including the soil type in each region, fertilization, irrigation, planting time (date) in each region, etc. Soil type, temperature and rainfall are different in different regions. Was the uniform management reasonable? The case should be clarified/justified.

In the section “Crop sampling and morphological measurements”:
The harvest date in each region must be specified precisely.

In the section “In situ rumen degradation”:
At what level was the feeding done? Maintenance?

In the Discussion:
Many parts of the discussion are similar to the review of sources. This part should be improved and the results should be interpreted more carefully and the causes of differences among the regions should be justified based on the characteristics and conditions of each region.

**Response:**

Thanks for your suggestions and we have carefully revised the relative contents in accordance with the comments.

1) In the section “Corn cultivars, growing regions, and crop production”:

The planting information as well regional temperature and precipitation were represented in text and Table 1, respectively. The planting time (date) was not mentioned because the samples were harvested at the same stage (kernel maturity stage of half milk line) whenever they were planted. The uniform management is unreasonable, so we suppled the sentence “The fertilization and irrigation were managed by the local farmers accordingly” in manuscript for better explanation. As for the relative information of soil, we are really SORRY for that. Because of some external factors, the collected soil samples were broken and could not be used anymore. We tried our best to measure, but the data was not ideal. Finally, we gave up the soil information to ensure the accuracy of data. We are eager for your understanding.

2) In the section “Crop sampling and morphological measurements”:

We must explain honestly for that. The specific harvest date for each region was ambiguous. But we promise that all samples were harvested from late September to early October at kernel maturity stage of half milk line.

3) In the section “In situ rumen degradation”:

The ingredients and nutrient contents of TMR are list in Table 2 of manuscript according to recommendations in NRC (2001) guidelines. We have added the reference in the new version, sorry for the careless.

Reference:

NRC. Nutrient requirements of dairy cattle. 7th rev ed. 2001. Natl Acad Sci, Washington, DC.

4) In the Discussion:

We totally agree with you, so some new references were inserted in the discussion section of this latest manuscript to discuss more depth.

Anyway, thank you again, and we all be happy to edit the text further based on the helpful comments from you!

**Reviewer #5:** The manuscript evaluates the variation of morphological characteristics, nutritional value, ruminal degradability, and molecular structural makeup of diverse whole-plant silage corn cultivars among different growing regions. The study suggested that the morphological characteristics, protein nutritional values and rumen degradability significantly varied among different grown regions due to distinguished molecular structures. The manuscript is novel and important for the readers.

**Response:**

Thanks for your affirmation, we would like to publish this article in “PLoS One” so much.
